# Alcohol consumption and non-Hodgkin lymphoma in a cohort of older women

**DOI:** 10.1038/sj.bjc.6690547

**Published:** 1999-07

**Authors:** B C-H Chiu, J R Cerhan, S M Gapstur, T A Sellers, W Zheng, C T Lutz, R B Wallace, J D Potter

**Affiliations:** Department of Preventive Medicine and Environmental Health, The University of lowa College of Medicine, lowa City, IA, 52242, USA; Department of Preventive Medicine, Northwestern University Medical School, Chicago, IL, 60611, USA; Department of Health Sciences Research, Mayo Clinic, Rochester, MN, 55905, USA; School of Public Health and Cancer Center, University of South Carolina, Columbia, SC, 29203, USA; Department of Pathology, The University of lowa College of Medicine, lowa City, IA, 52240, USA; Fred Hutchinson Cancer Research Center, Seattle, WA, 98104, USA

**Keywords:** lymphoma, alcohol, non-Hodgkin, cohort study

## Abstract

We investigated the relation of alcohol consumption to risk of non-Hodgkin's lymphoma (NHL) in a cohort of 35 156 lowa women aged 55–69 years who participated in the lowa Women's Health Study in 1986. Alcohol consumption at baseline was obtained using a mailed questionnaire. During the 9-year follow-up period, 143 incident cases of NHL were identified. Higher alcohol consumption was significantly associated with a decreased risk of NHL (*P*-trend = 0.03). Compared to non-drinkers, multivariate-adjusted relative risks (RRs) were decreased for women with intake of ≤ 3.4 g day^−1^ (RR = 0.78; 95% confidence interval (CI) 0.51–1.21) and > 3.4 g day^−1^ (RR = 0.59; 0.36–0.97). The inverse association could not be attributed to one particular type of alcoholic beverage, although red wine (RR = 0.21 for > 2 glasses per month vs non-drinker; 0.05–0.86; *P*-trend = 0.02) has the most distinct effect. The apparent protective effect was universal regardless of specific NHL grade or Working Formulation subtype, but was most pronounced for nodal NHL (RR = 0.48; 0.26–0.90; *P*-trend = 0.01) and low-grade NHL (RR = 0.52; 0.21–1.26; *P*-trend = 0.05). These data suggest that moderate alcohol consumption is inversely associated with the risk of NHL in older women and the amount of alcohol consumed, rather than the type of alcoholic beverages, appears to be the main effect determinant. © 1999 Cancer Research Campaign


*British Journal of Cancer* (1999) **80**(9), 1476–1482
© 1999 Cancer Research Campaign
Article no. bjoc.1998.0547

### Alcohol consumption and non-Hodgkin lymphoma in a cohort of older women

**BC-H Chiu[1,*], JR Cerhan[1], SM Gapstur[2], TA Sellers[3], W Zheng[4], CT Lutz[5], RB Wallace[1] and JD Potter[6]**

[1]Department of Preventive Medicine and Environmental Health, The University of Iowa College of Medicine, Iowa City, IA 52242, USA; [2]Department of Preventive Medicine, Northwestern University Medical School, Chicago, IL 60611, USA; [3]Department of Health Sciences Research, Mayo Clinic, Rochester, MN 55905, USA; [4]School of Public Health and Cancer Center, University of South Carolina, Columbia, SC 29203, USA; [5]Department of Pathology, The University of Iowa College of Medicine, Iowa City, IA 52240, USA; [6]Fred Hutchinson Cancer Research Center, Seattle, WA 98104, USA

**Summary** We investigated the relation of alcohol consumption to risk of non-Hodgkin's lymphoma (NHL) in a cohort of 35 156 Iowa women aged 55–69 years who participated in the Iowa Women's Health Study in 1986. Alcohol consumption at baseline was obtained using a mailed questionnaire. During the 9-year follow-up period, 143 incident cases of NHL were identified. Higher alcohol consumption was significantly associated with a decreased risk of NHL (*P*-trend = 0.03). Compared to non-drinkers, multivariate-adjusted relative risks (RRs) were decreased for women with intake of ≤ 3.4 g $day^{-1}$ (RR = 0.78; 95% confidence interval (CI) 0.51–1.21) and > 3.4 g $day^{-1}$ (RR = 0.59; 0.36–0.97). The inverse association could not be attributed to one particular type of alcoholic beverage, although red wine (RR = 0.21 for > 2 glasses per month vs non-drinker; 0.05–0.86; *P*-trend = 0.02) has the most distinct effect. The apparent protective effect was universal regardless of specific NHL grade or Working Formulation subtype, but was most pronounced for nodal NHL (RR = 0.48; 0.26–0.90; *P*-trend = 0.01) and low-grade NHL (RR = 0.52; 0.21–1.26; *P*-trend = 0.05). These data suggest that moderate alcohol consumption is inversely associated with the risk of NHL in older women and the amount of alcohol consumed, rather than the type of alcoholic beverages, appears to be the main effect determinant.

**Keywords:** lymphoma; alcohol; non-Hodgkin; cohort study

Non-Hodgkin's lymphoma (NHL) is a malignant cell infiltration of the lymphatic system. The incidence of NHL in the USA increased 81% between 1973 and 1994 (Ries et al, 1997), a percentage increase exceeded only by that for prostate cancer, lung cancer in women, and melanoma. The increase has occurred in both males and females, with older persons showing the most dramatic increases (Ballester et al, 1993; Weisenburger, 1994). In 1997, approximately 53 600 new cases of NHL would have been diagnosed in the USA, and over 30 000 deaths would be attributed to this cancer (Ries et al, 1997).

Risk factors for NHL are not well-understood. The best-established risk factors for NHL are immunosuppressed states due to primary immune deficiency disease (Filipovich et al, 1992) or acquired immune alterations (e.g. iatrogenic immuno-suppression; Sjögren's syndrome, human immunodeficiency virus) (Hoover, 1992). Other suggested risk factors include history of diabetes (Cerhan et al, 1997) or prior blood transfusion (Cerhan et al, 1993), certain dietary factors (Franceschi et al, 1989*b*; Ward et al, 1994; Chiu et al, 1996; Tavani et al, 1997; De Stefani et al, 1998), use of hair dyes (Cantor et al, 1988) and occupational exposure to pesticides, in particular, phenoxy herbicides and organophosphate insecticides (Zahm et al, 1993).

These are, at best, clues, and the aetiology of NHL, and particularly the causes of NHL in older persons, needs further research. Alcohol may be immunomodulatory as several host defense factors have been implicated in the increased incidence of infectious diseases observed in alcohol abusers (Mufti et al, 1989; MacGregor, 1986). However, the role of moderate alcohol use on the immune system is less clear (Mufti et al, 1989), and the role of moderate alcohol consumption in the aetiology of NHL has received only limited attention. Among eight reports from five case-control studies that have assessed this association, one found a significant positive association (De Stefani et al, 1998), five reported no association (Cartwright et al, 1988; Franceschi et al, 1989*a*, 1989*b*; Tavani et al, 1994, 1997), one showed a weak inverse association (Brown et al, 1992) and one found a significant inverse association (Nelson et al, 1997). To date, no cohort studies have evaluated this association. We therefore examined the relation of alcohol consumption to risk of NHL in the Iowa Women's Health Study (IWHS), a prospective study of post-menopausal women.

### METHODS

This study was approved by The Human Subjects Review Boards of the University of Minnesota and the University of Iowa. The Iowa Women's Health Study is a prospective cohort study of cancer in a sample of Iowa women aged 55–69 years selected from the State of Iowa automobile driver's licence list in 1985. The list represented approximately 94% of all Iowa women in this age range (Lynch et al, 1994). In January 1986, a questionnaire and a letter describing the purpose of the study were mailed to 98 030

*Received 20 October 1998*
*Revised 27 November 1998*
*Accepted 2 December 1998*

*Correspondence to:* JR Cerhan, Department of Health Sciences Research, Mayo Clinic, 200 First Street SW, Rochester, MN 55905, USA

*Present address: Department of Preventive and Societal Medicine, University of Nebraska Medical Center, Omaha, NE 68198, USA.

**Table 1** Age-adjusted relative risk of non-Hodgkin lymphoma among post-menopausal women according to participant characteristics, Iowa Women's Health Study, 1986–1994

| Variables | No. of cases[a] | Person-years | RR[b] | 95% CI[c] |
|---|---|---|---|---|
| Residence | | | | |
| Non-farm | 112 | 253854 | 1.00 | (referent) |
| Farm | 40 | 63730 | 1.47 | (1.02–2.11) |
| Education | | | | |
| < High school | 10 | 29015 | 1.00 | (referent) |
| High school | 91 | 166802 | 1.72 | (0.90–3.32) |
| > High school | 53 | 123495 | 1.34 | (0.68–2.65) |
| Marital status | | | | |
| Current | 106 | 244807 | 1.00 | (referent) |
| Former | 42 | 65075 | 1.33 | (0.93–1.92) |
| Never | 6 | 7675 | 1.65 | (0.72–3.76) |
| Diagnosis of diabetes | | | | |
| No | 137 | 299051 | 1.00 | (referent) |
| Yes | 16 | 18587 | 1.81 | (1.08–3.04) |
| Smoking status | | | | |
| Never | 99 | 210135 | 1.00 | (referent) |
| Former | 31 | 59807 | 1.14 | (0.76–1.70) |
| Current | 21 | 45253 | 1.06 | (0.66–1.70) |
| History of transfusion | | | | |
| Never | 96 | 227646 | 1.00 | (referent) |
| Ever | 54 | 80887 | 1.59 | (1.14–2.22) |
| Red meat (servings/month) | | | | |
| < 22 | 43 | 101714 | 1.00 | (referent) |
| 22–36 | 42 | 100812 | 1.01 | (0.66–1.55) |
| > 36 | 58 | 94487 | 1.50 | (1.01–2.23) |
| Fruits (servings/month) | | | | |
| < 54 | 62 | 99937 | 1.00 | (referent) |
| 54–84 | 37 | 99309 | 0.58 | (0.38–0.87) |
| > 85 | 44 | 97768 | 0.68 | (0.46–1.01) |

[a] Number of cases may not sum to 154 due to missing data. [b]RR = age-adjusted relative risk by 5-year groupings using the method of Mantel and Haenszel. [c]CI = confidence interval.

**Table 2** Baseline characteristics of the cohort according to level of alcohol intake, Iowa Women's Health Study, 1986–1994

| | Level of alcohol intake | | |
|---|---|---|---|
| Risk factor | Non-drinkers ($n$ = 19 283) | ≤ 3.4 g per day ($n$ = 7967) | > 3.4 g per day ($n$ = 7906) |
| Residence | | | |
| Non-farm | 77% | 81% | 88% |
| Farm | 23% | 19% | 12% |
| Marital status | | | |
| Current | 77% | 78% | 79% |
| Former | 21% | 20% | 19% |
| Never | 3% | 2% | 2% |
| Diagnosis of diabetes | | | |
| Never | 92% | 97% | 98% |
| Ever | 8% | 3% | 3% |
| History of blood transfusion | | | |
| Never | 73% | 74% | 75% |
| Ever | 27% | 26% | 25% |
| Animal protein, g per day | | | |
| Mean ± s.d. | 59 ± 26.8 | 60 ± 24.8 | 60 ± 25.3 |
| Median | 56 | 57 | 57 |
| Animal fat, g per day | | | |
| Mean ± s.d. | 39 ± 19.0 | 40 ± 18.4 | 40 ± 18.4 |
| Median | 36 | 37 | 37 |
| Red meat, servings per month | | | |
| Mean ± s.d. | 32 ± 22.0 | 32 ± 20.6 | 32 ± 20.7 |
| Median | 28 | 28 | 28 |
| All fruits, servings per month | | | |
| Mean ± s.d. | 77 ± 49.1 | 78 ± 46.9 | 69 ± 43.9 |
| Median | 70 | 70 | 62 |

randomly selected women, followed by reminder mailings 2 and 4 weeks later. The self-administered questionnaire included information on alcohol intake, diet assessment, weight history, health habits, medical history and family medical history. From the original sample, 41 837 completed questionnaires were returned for a response rate of 42.7%. There were only minor demographic differences between the respondents and non-respondents at baseline (Folsom et al, 1990). Compared with non-respondents, the respondents have had subsequently lower 5-year cancer incidence and mortality rates, mainly for smoking-related diseases (Bisgard et al, 1994).

Usual alcohol consumption was assessed as part of a semiquantitative food frequency questionnaire developed by Willett and colleagues (Willett et al, 1985). For each type of alcoholic beverage, a commonly used unit – glass, bottle, 'shot' – was specified. Participants were asked how often, on average, over the past year they had consumed that amount of each alcoholic beverage. There were nine possible responses, ranging from 'never or less than once per month' to 'six or more times per day' to define their frequency of consumption of that unit. Frequencies were recorded separately for red wine, white wine, beer and liquor. The daily intake, in grams, was then calculated by multiplying the frequency with which each beverage was consumed by the ethanol content of the specific beverage (10.8 g of ethanol per four-ounce glass of red or white wine; 13.2 g per bottle or can of beer; and 15.1 g per drink or shot of liquor) (Gapstur et al, 1993), using a database provided by Willett and colleagues. Average daily alcohol intake was calculated by summing the contribution from each type of alcoholic beverage. The semiquantitative food frequency questionnaire has been shown to be a valid and reliable method for assessing average daily alcohol consumption (Giovannucci et al, 1991). Munger and co-workers (Munger et al, 1992) also examined the accuracy and reproducibility of the questionnaire in this cohort. Pearson correlation coefficients of alcohol intake from the baseline questionnaire with a second and third questionnaire were 0.99 and 0.98 respectively.

### Follow-up of the cohort

Follow-up questionnaires were mailed in October 1987, August 1989 and June 1992 to update risk factor information and address changes. Deaths were ascertained by linkage to Iowa death certificates data and, for questionnaire non-responders and emigrants from Iowa, to the National Death Index. Approximately 4% of the cohort has been censored for migration out of Iowa. Unknown vital status for members of the cohort is estimated to be less than 1%.

Incident NHL in Iowa residents was ascertained through the State Health Registry of Iowa, part of the US National Cancer Institute's Surveillance, Epidemiology, and End Results (SEER) programme (Ries et al, 1997). Cohort participants were linked annually to the database with combination of first, last and maiden names, ZIP code, date of birth and Social Security number.

Topographic and morphologic data were coded using the *International Classification of Diseases for Oncology, Second Edition* (Percy et al, 1990). The histological subtypes of NHL were grouped according to the Working Formulation (Percy et al,

*© 1999 Cancer Research Campaign*

**Table 3** Age and multivariate relative risks[a] of non-Hodgkin lymphoma among Iowa women according to level of intake of various alcoholic beverages, Iowa Women's Health Study, 1986–1994

| Alcohol | Level of intake: Non-drinkers | ≤ Median | > Median | *P*-trend |
|---|---|---|---|---|
| Alcohol, g per day | | | | |
| Range | None | ≤ 3.4 | > 3.4 | |
| No. of cases | 96 | 27 | 20 | |
| Person-years | 163282 | 67492 | 66241 | |
| RR[a] (age/energy) | 1.00 | 0.69 | 0.53 | 0.005 |
| RR (full model) | 1.00 | 0.78 | 0.59 | 0.03 |
| 95% CI | (Referent) | (0.51–1.21) | (0.36–0.97) | |
| Beer, servings per month | | | | |
| Range | None | ≤ 4 | > 4 | |
| No. of cases | 96 | 9 | 7 | |
| Person-years | 163282 | 33665 | 19304 | |
| RR[a] (age/energy) | 1.00 | 0.48 | 0.66 | 0.06 |
| RR (full model) | 1.00 | 0.54 | 0.69 | 0.11 |
| 95% CI | (Referent) | (0.27–1.07) | (0.32–1.51) | |
| Red wine, servings per month | | | | |
| Range | None | ≤ 2 | > 2 | |
| No. of cases | 96 | 19 | 2 | |
| Person-years | 163282 | 44450 | 19336 | |
| RR (age/energy) | 1.00 | 0.75 | 0.18 | 0.007 |
| RR (full model) | 1.00 | 0.85 | 0.21 | 0.02 |
| 95% CI | (Referent) | (0.51–1.40) | (0.05–0.86) | |
| White wine, servings per month | | | | |
| Range | None | ≤ 2 | > 2 | |
| No. of cases | 96 | 21 | 4 | |
| Person-years | 163282 | 39344 | 24629 | |
| RR (age/energy) | 1.00 | 0.94 | 0.29 | 0.03 |
| RR (full model) | 1.00 | 1.07 | 0.35 | 0.09 |
| 95% CI | (Referent) | (0.66–1.73) | (0.13–0.96) | |
| Liquor, servings per month | | | | |
| Range | None | ≤ 4 | > 4 | |
| No. of cases | 96 | 15 | 10 | |
| Person-years | 163282 | 45475 | 31235 | |
| RR (age/energy) | 1.00 | 0.59 | 0.56 | 0.02 |
| RR (full model) | 1.00 | 0.65 | 0.61 | 0.06 |
| 95% CI | (Referent) | (0.37–1.13) | (0.32–1.19) | |

[a]Relative risks (RR) and 95% confidence intervals (CI), adjusted for age (continuous) and total energy (continuous) or (RR full model) age (continuous), residence (farm, non-farm), education (< high school, high school, > high school), marital status (current, former, or never), transfusion history (ever, never), diabetes history (yes, no), intake of red meat (< 22, 22–36, > 36 servings per month), intake of fruits (< 54, 54–84, > 84 servings per month) and total energy intake (continuous).

1990) into the following subtypes: small-cell lymphocytic lymphoma (ICD-O code 9670), follicular lymphoma (ICD-O codes 9690, 9691, 9693, 9695, 9696 and 9698), diffuse lymphoma (ICD-O codes 9672, 9673, 9675, 9680–9682, 9684 and 9686), and all other (ICD-O codes 9590, 9671 and 9700). In addition, NHL was subgrouped into three major categories by grade: low-grade (ICD-O codes 9670, 9671, 9691, 9693, 9695 and 9696), intermediate grade (ICD-O codes 9672, 9673, 9675, 9680–9682 and 9698) and other NHL (ICD-O codes 9684–9686, high-grade; and 9590, 9690, 9700, unclassified). NHL was also grouped as nodal or extra-nodal according to the primary site.

### Data analysis

Before data analysis, we excluded women with a self-reported history of cancer or prior use of cancer chemotherapy at the 1986 baseline survey ($n$ = 3903) to provide a cancer-free at-risk cohort. In addition, we excluded those who left 30 or more food items blank on the food frequency questionnaire or who had implausible daily energy intakes (i.e. < 600 Kcal or > 5000 Kcal; $n$ = 2778). This reduced the at-risk cohort to 35 156 women, among whom 143 incident cases of NHL developed.

Person-years of follow-up for each woman was accumulated from the date of the 1986 baseline questionnaire to one of the following events: (1) date of NHL diagnosis; (2) date of death (if in Iowa); (3) date of a move out of Iowa (if known); (4) the midpoint between the date of last contact and date located outside of Iowa (if date of move was unknown); (5) midpoint between date of last contact and date of death (for non-Iowa deaths). If none of these events occurred, follow-up was through 31 December 1994.

Women were classified *a priori* according to three levels of alcoholic beverage consumption: non-drinkers were defined as women whose reported total alcohol use was zero; and two other categories were based on a median split of drinkers for each type of alcoholic beverages (for total alcohol, 3.4 g per day; for beer and liquor, 4 servings per month; and for red wine and white wine, 2 servings per month). Of the responders to the baseline questionnaire, 3.8% of women left all four alcoholic beverages blank on

 *© 1999 Cancer Research Campaign*

**Table 4** Multivariate relative risks of subtypes of non-Hodgkin lymphoma among Iowa women according to tertile of intake of various alcoholic beverages, Iowa Women's Health Study, 1986–1994

| Subtypes and level of intake | Alcohol | Beer | Red wine | White wine | Liquor |
|---|---|---|---|---|---|
| NHL, nodal versus extranodal | | | | | |
| Nodal (*n* = 97)[a] | | | | | |
| None | 1.00 (Referent) | 1.00 (Referent) | 1.00 (Referent) | 1.00 (Referent) | 1.00 (Referent) |
| ≤ Median | 0.71 (0.42–1.20) | 0.58 (0.26–1.26) | 0.86 (0.48–1.55) | 0.82 (0.44–1.54) | 0.60 (0.31–1.16) |
| > Median | 0.48 (0.26–0.90) | 0.41 (0.13–1.31) | 0.29 (0.07–1.20) | 0.23 (0.06–0.97) | 0.51 (0.22–1.19) |
| *P* for trend | 0.01 | 0.05 | 0.08 | 0.03 | 0.04 |
| Extra-nodal (*n* = 41)[a] | | | | | |
| None | 1.00 (Referent) | 1.00 (Referent) | 1.00 (Referent) | 1.00 (Referent) | 1.00 (Referent) |
| ≤ Median | 1.00 (0.46–2.16) | 0.43 (0.10–1.83) | 0.81 (0.30–2.15) | 1.77 (0.80–3.88) | 0.79 (0.30–2.09) |
| > Median | 0.89 (0.39–2.02) | 1.45 (0.49–4.33) | – (–) | 0.69 (0.16–2.99) | 0.87 (0.30–2.54) |
| *P* for trend | 0.78 | 0.95 | 0.14 | 0.80 | 0.69 |
| NHL by grade | | | | | |
| Low (*n* = 43)[a] | | | | | |
| None | 1.00 (Referent) | 1.00 (Referent) | 1.00 (Referent) | 1.00 (Referent) | 1.00 (Referent) |
| ≤ Median | 0.33 (0.12–0.92) | 0.99 (0.41–2.40) | 0.61 (0.24–1.58) | 0.56 (0.20–1.61) | 0.49 (0.17–1.40) |
| > Median | 0.52 (0.21–1.26) | – (–) | – (–) | 0.48 (0.11–2.04) | 0.73 (0.25–2.09) |
| *P* for trend | 0.05 | 0.13 | 0.05 | 0.18 | 0.28 |
| Intermediate (*n* = 72)[a] | | | | | |
| None | 1.00 (Referent) | 1.00 (Referent) | 1.00 (Referent) | 1.00 (Referent) | 1.00 (Referent) |
| ≤ Median | 0.85 (0.47–1.54) | 0.24 (0.06–1.00) | 0.88 (0.44–1.77) | 1.26 (0.67–2.36) | 0.59 (0.26–1.32) |
| > Median | 0.67 (0.35–1.28) | 1.23 (0.51–2.97) | 0.41 (0.10–1.68) | 0.16 (0.02–1.19) | 0.58 (0.23–1.47) |
| *P* for trend | 0.21 | 0.64 | 0.21 | 0.16 | 0.12 |
| All Other (*n* = 21)[a,b] | | | | | |
| None | 1.00 (Referent) | 1.00 (Referent) | 1.00 (Referent) | 1.00 (Referent) | 1.00 (Referent) |
| ≤ Median | 1.67 (0.65–4.30) | 0.44 (0.06–3.43) | 1.56 (0.49–4.94) | 1.48 (0.41–5.39) | 1.40 (0.45–4.41) |
| > Median | 0.50 (0.11–2.26) | 0.64 (0.08–5.05) | – (–) | 0.98 (0.12–7.81) | 0.50 (0.06–3.95) |
| *P* for trend | 0.65 | 0.48 | 0.78 | 0.78 | 0.76 |
| NHL by subtype | | | | | |
| Small lymphocytic (*n* = 15)[a] | | | | | |
| None | 1.00 (Referent) | 1.00 (Referent) | 1.00 (Referent) | 1.00 (Referent) | 1.00 (Referent) |
| ≤ Median | 0.23 (0.03–1.74) | 1.36 (0.38–4.89) | 0.73 (0.16–3.35) | 0.44 (0.06–3.42) | 0.34 (0.04–2.67) |
| > Median | 0.48 (0.10–2.18) | – (–) | – (–) | 0.78 (0.10–6.25) | 0.52 (0.07–4.08) |
| *P* for trend | 0.18 | 0.47 | 0.30 | 0.57 | 0.32 |
| Follicular (*n* = 22)[a] | | | | | |
| None | 1.00 (Referent) | 1.00 (Referent) | 1.00 (Referent) | 1.00 (Referent) | 1.00 (Referent) |
| ≤ Median | 0.48 (0.14–1.65) | 0.65 (0.15–2.86) | 0.50 (0.11–2.19) | 0.57 (0.13–2.53) | 0.24 (0.03–1.81) |
| > Median | 0.34 (0.08–1.50) | – (–) | – (–) | – (–) | 0.70 (0.16–3.13) |
| *P* for trend | 0.09 | 0.18 | 0.14 | 0.14 | 0.29 |
| Diffuse (*n* = 72)[a] | | | | | |
| None | 1.00 (Referent) | 1.00 (Referent) | 1.00 (Referent) | 1.00 (Referent) | 1.00 (Referent) |
| ≤ Median | 0.94 (0.53–1.67) | 0.25 (0.06–1.03) | 1.01 (0.52–1.97) | 1.29 (0.69–2.43) | 0.70 (0.33–1.49) |
| > Median | 0.69 (0.36–1.33) | 1.25 (0.52–3.03) | 0.42 (0.10–1.76) | 0.17 (0.02–1.21) | 0.60 (0.23–1.52) |
| *P* for trend | 0.28 | 0.68 | 0.31 | 0.18 | 0.18 |
| All Other (*n* = 27)[a] | | | | | |
| None | 1.00 (Referent) | 1.00 (Referent) | 1.00 (Referent) | 1.00 (Referent) | 1.00 (Referent) |
| ≤ Median | 0.97 (0.38–2.48) | 0.63 (0.14–2.74) | 0.91 (0.30–2.76) | 1.14 (0.38–3.49) | 1.19 (0.43–3.26) |
| > Median | 0.66 (0.22–2.02) | 0.51 (0.07–3.93) | – (–) | 1.03 (0.23–4.63) | 0.69 (0.16–3.05) |
| *P* for trend | 0.51 | 0.40 | 0.26 | 0.88 | 0.78 |

[a]Adjusted for age (continuous), residence (farm, non-farm), education (< high school, high school, > high school) marital status (current, former, or never), transfusion history (ever, never), diabetes history (yes, no), intake of red meat (< 22, 22–36, > 36 servings per month), intake of fruits (< 54, 54–84, > 84 servings per month) and total energy intake (continuous). [b]Includes high-grade and unclassified NHL.

the food frequency questionnaire. Total daily alcohol intake for these women was considered to be zero grams per day for purpose of analysis. In a previous study of alcohol consumption and endometrial cancer in this cohort, it was found that when analyses were repeated with alcohol intake considered missing for these women, the results were comparable (Gapstur et al, 1993).

Non-dietary variables of interest were categorized into natural categories. Important dietary risk variables, including animal fat, animal protein, red meat, fruits and hamburger (Chiu et al, 1996), were categorized by tertiles based on the distribution of consumption in all respondents. All models with dietary factors were adjusted for total energy intake, either by including total energy as a covariate in the model (for analyses involving micronutrients and food groups) or by using the residual method for adjustment of macronutrients (Willett and Stampfer, 1986). The incidence rate of NHL in each category of exposure was calculated by dividing the number of NHL cases by the corresponding person-years of follow-up. The relative risk (RR) and 95% confidence intervals (CIs) were

© 1999 Cancer Research Campaign 

used as the measure of association. The Mantel–Haenszel procedure (Breslow and Day, 1987) was used to estimate age-adjusted RRs, and Cox proportional hazards regression (Cox, 1972) was used to estimate multivariate-adjusted RRs. Farm residence (Folsom et al, 1996), marital status, diet (high consumption of red meat and low consumption of fruits) (Chiu et al, 1996), diabetes history (Cerhan et al, 1997) and blood transfusion history (Cerhan et al, 1993) were all assessed as potential confounders. Proportion hazards assumption was tested and was not violated. Analyses were conducted using SAS (SAS Institute, Cary, NC, USA) software programs. Reported *P*-values are two-sided.

### RESULTS

During the 320 184 person-years of follow-up over the 9-year period, a total of 143 women developed NHL. In order to evaluate possible confounding, the association between the incidence of NHL and several potential risk factors were examined (Table 1). There were no clear associations between the incidence of NHL and education and smoking status at baseline. However, living on a farm, being formerly or never married, a history of diabetes mellitus, a history of blood transfusion, and higher consumption of red meat were positively associated with NHL; higher consumption of fruits was inversely associated with NHL.

The distribution of significant risk factors in Table 1, along with specific dietary factors that have been previously reported as risk factors for NHL in this cohort, were further evaluated according to level of alcohol consumption (Table 2). Women who lived on a farm consumed less alcohol compared to women who did not live on a farm. Women with a higher level of alcohol intake were more likely to be currently married and were less likely to have been diagnosed with diabetes mellitus. Intakes of animal protein, animal fat, red meat and hamburger showed little difference across levels of alcohol intake. However, women who consumed > 3.4 g of alcohol a day had a lower intake of fruits than those with lower levels of alcohol consumption.

Age- and energy-adjusted RRs and multivariate-adjusted RRs for NHL according to levels of intake of different alcoholic beverages are presented in Table 3, with the non-drinkers of all alcohol representing the reference category for each specific type of alcoholic beverage. All RRs discussed in the text compare the highest users to the non-drinkers. Inclusion of dietary and non-dietary NHL risk factors in multivariate Cox proportional hazards regression models generally yielded slightly weaker RR estimates for NHL compared with the age- and energy-adjusted values. Table 3 shows that alcohol consumption was inversely associated with risk of NHL (*P*-trend = 0.03). Compared to non-users, multivariate-adjusted relative risks of NHL were moderately decreased for women with average daily alcohol consumption of ≤ 3.4 g (RR = 0.78; 95% CI 0.51–1.21) but significantly decreased for average daily consumption greater than 3.4 g (RR = 0.59; 95% CI 0.36–0.97). Evidence for an inverse association persisted in the multivariate analysis even when we used a higher cut-point (> 10 g per day) or treated alcohol consumption as a continuous variable in the model (data not shown).

To determine whether the effect of alcohol consumption was related to specific types of alcoholic beverages, we examined the separate effects of beer, red wine, white wine and liquor. We found that the inverse association with alcohol could not be attributed to one particular type of alcohol-containing beverage, although intake of red wine showed the strongest protective effect. Compared to non-users, multivariate-adjusted relative risks of NHL for women whose average monthly intake of red wine was ≤ 2 servings and > 2 servings were 0.85 (0.51–1.40) and 0.21 (0.05–0.86) respectively. The inverse association was also apparent for beer, white wine and liquor. Compared with women who had not used any alcohol, the multivariate-adjusted RRs of NHL for women with higher than median intake were 0.69 (0.32–1.51) for beer, 0.35 (0.13–0.96) for white wine and 0.61 (0.32–1.19) for liquor. We also attempted to simultaneously adjust for each type of alcoholic beverage but were not able to fit a model, probably due likely to the highly correlated nature of specific types of alcohol.

We were also interested in whether alcohol consumption was associated with specific subtypes of NHL (Table 4). The inverse association was stronger for nodal NHL compared to extra-nodal NHL. Risk of developing nodal NHL decreased from 0.71 (0.42–1.20) for women with intakes of ≤ 3.4 g per day (compared to non-users) to 0.48 (0.26–0.90) for women with intakes of > 3.4 g per day (*P*-trend = 0.01). The corresponding RRs for extra-nodal NHL were 1.00 (0.46–2.16) and 0.89 (0.39–2.02) respectively.

When NHL was stratified by grade, inverse associations with all alcohol and specific types of alcohol were seen for low and intermediate grade tumours, although few estimates were statistically significant. For NHL subtypes, we observed an inverse dose–response association between alcohol consumption and all NHL subtypes, and specific types of alcohol generally showed similar trends to those seen for total alcohol use. However, most confidence limits included 1.0 and no test for trend reached statistical significance, perhaps due to the limited sample size in these analyses.

### DISCUSSION

We observed a statistically significant inverse association between alcohol consumption and NHL, with a 22% and 40% decreased risk for women with intake of alcohol ≤ 3.4 g per day and > 3.4 g per day, respectively, compared to women who do not drink alcohol. The inverse association with alcohol consumption could not be attributed to one particular type of alcoholic beverages, although red wine appeared to have the strongest association. The apparent inverse association with alcohol was somewhat stronger for nodal NHL and low-grade NHL, but most point estimates for alcohol use were below one for all grades and subtypes of NHL.

To our knowledge, this is the first prospective cohort study to examine the association between risk of NHL and use of alcoholic beverages. Among a handful of case-control studies that have addressed this issue, a positive association with beer consumption in men was reported in one hospital-based study (De Stefani et al, 1998), no association was reported in three hospital-based studies (Cartwright et al, 1988; Franceschi et al, 1989*a*, 1989*b*; Tavani et al, 1994, 1997), whereas a decreased risk was observed in two population-based studies (Brown et al, 1992; Nelson et al, 1997). In the only other study that has separated NHL into specific entities, Brown and colleagues (Brown et al, 1992) found that, although not statistically significant, the odds ratios for drinkers of any type of alcohol were less than 1.0 for NHL and for all subtypes except diffuse lymphoma. Nelson et al (1997) in a population-based case-control study found that the risk of NHL among women decreased significantly with increased consumption of alcoholic beverages, with risk 50% lower among those consuming five or more drinks per week compared to non-drinkers. In that

 © 1999 Cancer Research Campaign study, a statistically significant inverse association was not observed in men, although point estimates for each type of alcoholic beverage were similar to those for women. Our finding of an inverse association with alcohol consumption is thus in line with results from population-based case-control studies (Brown et al, 1992; Nelson et al, 1997). Hospital-based case-control studies of alcohol are difficult to interpret due to the over representation of alcohol use among hospital patients.

Since the epidemiologic data relating NHL to alcohol intake are sparse, biologic mechanisms have not been well explored to date. Although high alcohol ingestion, either episodically or on a regular basis, is regarded as immunosuppressive (MacGregor, 1986; Mufti et al, 1989), less is known about the immunomodulation effects of light-to-moderate alcohol consumption (i.e. social drinking). Moderate, social alcohol use could potentially either suppress or enhance the induction and growth of cancers through its effect on membrane fluidity or composition (Freund, 1979; Mufti et al, 1989). The effect of altered membrane composition on lymphocytes remains to be determined. Social drinking has been shown to impair lymphokine-activated killer activity, but not natural killer activity (Bounds et al, 1994). However, the relation of alcohol consumption in the context of social drinking to the immune system and the development of NHL is not known.

Consumption of alcoholic beverages might be linked to lower risk of NHL through its effect on insulin levels. A series of studies have shown that both insulin-mediated and non-insulin mediated glucose disposal might be enhanced in individuals consuming light-to-moderate amounts of alcohol (Stampfer et al, 1988; Facchini et al, 1994; Rimm et al, 1995; Bell, 1996; Lazarus et al, 1997). Moderate alcohol consumption among healthy people has been associated with increased insulin sensitivity and a reduced risk of diabetes (Stampfer et al, 1988; Rimm et al, 1995). As diabetes mellitus has been previously associated with NHL in some studies (Ragozzino et al, 1982; Natazuka et al, 1994; Cerhan et al, 1997), it is biologically plausible that moderate alcohol intake might be indirectly related to lower risk of NHL through its effect on increased insulin sensitivity and glucose metabolism. However, the alcohol association remained after adjustment for history of diabetes in these data.

The effects of light-to-moderate alcohol with various concentrations of dietary nutrients in immunomodulation in humans is also poorly understood. Only two (Franceschi et al, 1989*b*; Tavani et al, 1997) previous epidemiologic studies of alcohol intake and NHL have collected information on dietary intake. It is also possible that the protective association may be attributable to antioxidant micronutrients. Consumption of red wine can significantly increase the serum antioxidant capacity (Whitehead et al, 1995). Low serum levels of antioxidant micronutrients have been associated with childhood leukaemia and lymphoma (Malvy et al, 1993).

Consideration must be given to the potential limitations in the present study that may have led to the observed inverse association. First, alcohol consumption was assessed using a food-frequency questionnaire that asked about average intake over the last year. Changes in alcohol consumption throughout life may lead to random mis-classification that contributed a bias toward a finding of no association. Systematic error may also be present because it is possible that heavy drinkers underreported their alcohol consumption. The group of non-drinkers or light drinkers may include some former heavy drinkers (Lazarus et al, 1997). This has been identified as a potential source of bias in studies of alcohol and cardiovascular disease (Shaper et al, 1988). Unfortunately, our data were not able to address this issue. Finally, it remains possible that health status or certain medical conditions may lead to restriction of alcohol intake, although that, too, could reduce the likelihood of seeing an increased association with higher alcohol intake.

On balance, however, our study has several advantages over previous case-control studies of alcohol intake and NHL. The prospective design avoids the possibility of biased recall of alcohol consumption. Other strengths include the use of a large, well-defined sample derived from a general population and case ascertainment using a SEER tumour registry.

In conclusion, our data suggest that light-to-moderate alcohol consumption is associated with the risk of NHL in older women. The present study also suggests that the amount of alcohol consumed, rather than the type of alcohol-containing beverage, is the main effect determinant. Our finding that moderate alcohol consumption might be protective against NHL is intriguing, but requires further research. Future studies should critically evaluate dose of alcohol ingestion and lifetime pattern of alcohol intake.

### ACKNOWLEDGEMENTS

The authors thank Kathleen McKeen, State Health Registry of Iowa, and Ching-Ping Hong, MS, University of Minnesota, for their helpful contributions. This research was supported by National Cancer Institute Grant R01 CA39742. Dr Cerhan was supported in part by a National Cancer Institute Preventive Oncology Academic Award (K07 CA64220).

### REFERENCES

Ballester OF, Moscinski L, Spiers A and Balducci L (1993) Non-Hodgkin's lymphoma in the older person: a review. *J Am Geriatr Soc* **41**: 1245–1254

Bell DS (1996) Alcohol and the NIDDM patient. *Diabetes Care* **19**: 509–513

Bisgard KM, Folsom AR, Hong CP and Sellers TA (1994) Mortality and cancer rates in nonrespondents to a prospective cohort study of older women: 5-year follow-up. *Am J Epidemiol* **139**: 990–1000

Bounds W, Betzing KW, Stewart RM and Holcombe RF (1994) Social drinking and the immune response: impairment of lymphokine-activated killer activity. *Am J Med Sci* **307**: 391–395

Breslow NE and Day NE (1987) *Statistical Methods in Cancer Research. Vol. II. The Design and Analysis of Cohort Studies*. International Agency for Research on Cancer Scientific Publication No. 88. IARC: Lyon

Brown LM, Gibson R, Burmeister LF, Schuman LM, Everett GD and Blair A (1992) Alcohol consumption and risk of leukemia, non-Hodgkin's lymphoma, and multiple myeloma. *Leukemia Res* **16**: 979–984

Cantor KP, Blair A, Everett G, Van Lier S, Burmeister L, Dick FR, Gibson RW and Schuman L (1988) Hair dye use and risk of leukemia and lymphoma. *Am J Public Health* **78**: 570–571

Cartwright RA, McKinney PA, O'Brien C, Richards IDG, Roberts B, Lauder I, Darwin, CM, Bernard SM and Bird CC (1988) Non-Hodgkin's lymphoma: case control epidemiological study in Yorkshire. *Leukemia Res* **12**: 81–88

Cerhan JR, Wallace RB, Folsom AR, Potter JD, Munger RG and Prineas RJ (1993) Transfusion history and cancer risk in older women. *Ann Intern Med* **119**: 8–15

Cerhan JR, Wallace RB, Folsom AR, Potter JD, Sellers TA, Zheng W and Lutz CT (1997) Medical history risk factors for non-Hodgkin's lymphoma in older women. *J Natl Cancer Inst* **89**: 314–318

Chiu BC-H, Cerhan JR, Folsom AR, Sellers TA, Kushi LH, Wallace RB, Zheng W and Potter JD (1996) Diet and risk of non-Hodgkin lymphoma in older women. *JAMA* **275**: 1315–1321

Cox DR (1972) Regression models and life tables (with discussions). *J R Stat Soc B* **34**: 187–220

De Stefani E, Fierro L, Barrios E and Ronco A (1998) Tobacco, alcohol, diet and risk of non-Hodgkin's lymphoma: a case-control study in Uruguay. *Leukemia Res* **22**: 445–452

Facchini F, Chen Y-DI and Reaven GM (1994) Light-to-moderate alcohol intake is associated with enhanced insulin sensitivity. *Diabetes Care* **17**: 115–119

*© 1999 Cancer Research Campaign*

Filipovich AH, Mathur A, Kamat D and Shapiro RS (1992) Primary immunodeficiencies: genetic risk factors for lymphoma. *Cancer Res* **52**: 5465s–5467s

Folsom AR, Kaye SA, Prineas RJ, Potter JD, Gapstur SM and Wallace RB (1990) Increased incidence of carcinoma of the breast associated with abdominal adiposity in postmenopausal women. *Am J Epidemiol* **131**: 794–803

Folsom AR, Zhang S, Sellers TA, Zheng W, Kushi LH and Cerhan JR (1996) Cancer incidence among women living on farms: findings from the Iowa Women's Health Study. *J Occup Environ Med* **38**: 1171–1176

Franceschi S, Serraino D, Bidoli E, Talamini R, Tirelli U, Carbone A and La Vecchia C (1989*a*) The epidemiology of non-Hodgkin's lymphoma in the north-east of Italy: a hospital-based case-control study. *Leukemia Res* **13**: 465–472

Franceschi S, Serraino D, Carbone A, Talamini R and La Vecchia C (1989*b*) Dietary factors and non-Hodgkin's lymphoma: a case-control study in the northeastern part of Italy. *Nutr Cancer* **12**: 333–341

Freund G (1979) Possible relationships of alcohol in membranes to cancer. *Cancer Res* **39**: 2899–2901

Gapstur SM, Potter JD, Sellers TA, Kushi LH and Folsom AR (1993) Alcohol consumption and postmenopausal endometrial cancer: results from the Iowa Women's Health Study. *Cancer Causes Control* **4**: 323–329

Giovannucci E, Colditz G, Stampfer MJ, Rimm EB, Litin L, Sampson L and Willett WC (1991) The assessment of alcohol consumption by a simple self-administered questionnaire. *Am J Epidemiol* **133**: 810–817

Hoover RN (1992) Lymphoma risks in populations with talered immunity to – a search for a mechanism. *Cancer Res* **52**: 5477s–5478s

Lazarus R, Sparrow D and Weiss ST (1997) Alcohol intake and insulin levels. The Normative Aging Study. *Am J Epidemiol* **145**: 909–916

Lynch CF, Logsden-Sackett N, Edwards SL and Cantor KP (1994) The driver's license list as a population-based sampling frame in Iowa. *Am J Public Health* **84**: 469–472

MacGregor RR (1986) Alcohol and immune defense. *JAMA* **256**: 1474–1479

Malvy DJ, Burtschy B, Arnaud J, Sommelet D, Laverger G, Dostalova L, Drucker J and Amedee-Manesme O (1993) Serum beta-carotene and antioxidant micronutrients in children with cancer: the Cancer in Children and Antioxidant Micronutrients French Study Group. *Int J Epidemiol* **22**: 761–771

Mufti SI, Darban HR and Watson RR (1989) Alcohol, cancer, and immunomodulation. *Critical Rev Oncol Hematol* **9**: 243–261.

Munger RG, Folsom AR, Kushi LH, Kaye SA and Sellers TA (1992) Dietary assessment of older Iowa women with a food-frequency questionnaire: nutrient intake, reproducibility, and comparison to 24-hour dietary recall interviews. *Am J Epidemiol* **136**: 192–200

Natazuka T, Manabe Y, Kono M, Murayama T, Matsui T and Chihara K (1994) Association between non-insulin dependent diabetes mellitus and non-Hodgkin's lymphoma. *Br Med J* **309**: 1269

Nelson RA, Levine AM, Marks G and Bernstein L (1997) Alcohol, tobacco and recreational drug use and the risk of non-Hodgkin's lymphoma. *Br J Cancer* **76**: 1532–1537

Percy C, Holten V and Muir C (1990) *International Classification of Diseases for Oncology*, 2nd Edn. World Health Organization: Geneva

Ragozzino M, Melton LJ, Chu CP and Palumbo PJ (1982) Subsequent cancer risk in the incidence cohort of Rochester, Minnesota, residents with diabetes mellitus. *J Chronic Dis* **35**: 13–9

Ries LAG, Kosary CL, Hankey BF, Miller BA, Harras A and Edwards BK (1997) SEER cancer statistics review, 1973–1994. NIH Pub. No. 97-2789. National Cancer Institute: Bethesda, MD.

Rimm EB, Chan J, Stampfer MJ, Colditz GA and Willett WC (1995) Prospective study of cigarette smoking, alcohol use, and the risk of diabetes in men. *Br Med J* **310**: 555–559

Shaper A, Wannamethee G and Walker M (1988) Alcohol and mortality in British men: explaining the U-shaped curve. *Lancet* **2**: 1267–1273

Stampfer MJ, Colditz GA, Willett WC, Manson JE, Arky RA, Hennekens CH and Speizer FE (1988) A prospective study of moderate alcohol drinking and risk of diabetes in women. *Am J Epidemiol* **128**: 549–558

Tavani A, Negri E, Franceschi S, Serraino D and La Vecchia C (1994) Smoking habits and non-Hodgkin's lymphoma: a case-control study in northern Italy. *Prev Med* **23**: 447–452

Tavani A, Pregnolato A, Negri E, Franceschi S, Serraino D, Carbone A and La Vecchia C (1997) Diet and risk of lymphoid neoplasms and soft tissue sarcomas. *Nutr Cancer* **27**: 256–260

Ward MH, Zahm SH, Weisenburger DD, Gridley G, Cantor KP, Saal RCF and Blair A (1994) Dietary factors and non-Hodgkin's lymphoma in Nebraska (United States). *Cancer Causes Control* **5**: 422–432

Weisenburger DD (1994) Epidemiology of non-Hodgkin's lymphoma: recent findings regarding an emerging epidemic. *Ann Oncol* **5**: s19–24

Whitehead TP, Robinson D, Allaway S, Syms J and Hale A (1995) Effect of red wine ingestion on the antioxidant capacity of serum. *Clin Chem* **41/1**: 32–35

Willett WC and Stampfer MJ (1986) Total energy intake: implications for epidemiologic analyses. *Am J Epidemiol* **124**: 17–27

Willett WC Sampson L, Stampfer MJ, Rosner B, Bain C, Witschi J, Hennekens CH and Speizer FE (1985) Reproducibility and validity of a semiquantitative food frequency questionnaire. *Am J Epidemiol* **122**: 51–65

Zahm SH, Weisenburger DD, Saal RC, Vaught JB, Babbitt PA and Blair A (1993) The role of agricultural pesticide use in the development of non-Hodgkin's lymphoma in women. *Arch Environ Health* **48**: 353–358

*© 1999 Cancer Research Campaign*
